# Creating a National Specimen Referral System in Guinea: Lessons From Initial Development and Implementation

**DOI:** 10.3389/fpubh.2019.00083

**Published:** 2019-04-16

**Authors:** Claire J. Standley, Rigo Muhayangabo, Mamadou S. Bah, Alpha M. Barry, Ebi Bile, Julie E. Fischer, Will Heegaard, Lamine Koivogui, Said K. Lakiss, Erin M. Sorrell, Amanda VanSteelandt, Anicet G. Dahourou, Lise D. Martel

**Affiliations:** ^1^Center for Global Health Science and Security, Georgetown University, Washington, DC, United States; ^2^International Medical Corps, Conakry, Guinea; ^3^Ministry of Health, Conakry, Guinea; ^4^US Centers for Disease Control and Prevention, Atlanta, GA, United States

**Keywords:** laboratory systems, Guinea, specimen referral, disease diagnostics, health systems strengthening

## Abstract

In the wake of the 2014–2016, West Africa Ebola virus disease (EVD) outbreak, the Government of Guinea recognized an opportunity to strengthen its national laboratory system, incorporating capacity and investments developed during the response. The Ministry of Health (MOH) identified creation of a holistic, safe, secure, and timely national specimen referral system as a priority for improved detection and confirmation of priority diseases, in line with national Integrated Disease Surveillance and Response guidelines. The project consisted of two parts, each led by different implementing partners working collaboratively together and with the Ministry of Health: the development and approval of a national specimen referral policy, and pilot implementation of a specimen referral system, modeled on the policy, in three prefectures. This paper describes the successful execution of the project, highlighting the opportunities and challenges of building sustainable health systems capacity during and after public health emergencies, and provides lessons learned for strengthening national capabilities for surveillance and disease diagnosis.

## Introduction

Laboratories are critical infrastructure for early detection and reporting of disease, and are most effective when organized into an integrated, multi-level network, enabling timely access to appropriate diagnostic tools at each level (1, 2). Tiered laboratory networks facilitate sequential diagnostic testing to identify or confirm the etiological agent(s) causing disease. Within the laboratory network, a formal structure for the referral and transport of diagnostic specimens can minimize transfer steps and facilitate rapid diagnosis and laboratory confirmation, thus reducing the time for reporting of new cases, or an emerging outbreak, as well as improving safe and secure sample management (3–6).

The Republic of Guinea did not have a national system for referral of diagnostic specimens prior to the emergence of Ebola virus disease (EVD) in its southeastern Forest Region in late 2014. Mechanisms for transport of samples from the peripheral parts of the health system to the national level were limited to vertically-funded disease control programs, such as those managed by the World Health Organization (WHO) in collaboration with the Ministry of Health (MOH) for vaccine-preventable illnesses (7). Although the transport system for vaccine-preventable illnesses, for example, encapsulated multiple diseases such as polio and measles, it was not designed to cover all notifiable or even priority diseases and was fully supported by external funding. During the EVD outbreak in 2014–2016, an *ad hoc* system emerged to cope with the urgent need to refer and transport large numbers of diagnostic specimens between Ebola Treatment Units and specialized, often temporary, laboratories set up to deal with the crisis. Significant investments, including training, vehicles, and materials, were provided down to the local level to support rapid testing of suspected EVD cases. A number of international donors, including the United States, France, and Russia, provided substantial assistance with respect to laboratory capacity building, including provision of self-contained laboratory units that were later donated to the Government of Guinea (8–12). As the outbreak declined in intensity, the MOH identified an opportunity to leverage investments made during the crisis to create a comprehensive and holistic national system for specimen referral.

Based on this experience, and in the context of broader efforts to strengthen the national laboratory network, the MOH requested assistance from international partners to develop a national specimen referral system, which would cover all priority diseases requiring confirmatory diagnosis. The system needed to (1) remain consistent with national disease detection and surveillance guidelines, such as those developed under Integrated Disease Surveillance and Response (IDSR; known in Guinea by its French acronym, SIMR); (2) align with international frameworks, such as the International Health Regulations (IHR 2005); and (3) consider local constraints, such as long-term availability of resources, prospective vs. actual capacity at the different levels of the laboratory system, and sustainable transport mechanisms. This effort was initiated by the MOH in collaboration with CDC's Guinea country office and its implementing partners at Georgetown University^1^ (GU) and International Medical Corps (IMC). Here, we present an overview of the project, with an emphasis on specific next steps for Guinea's system of specimen referral, as well as broader lessons learned for developing national capacity on the back of a public health crisis.

## Methods

**Figure 1:**
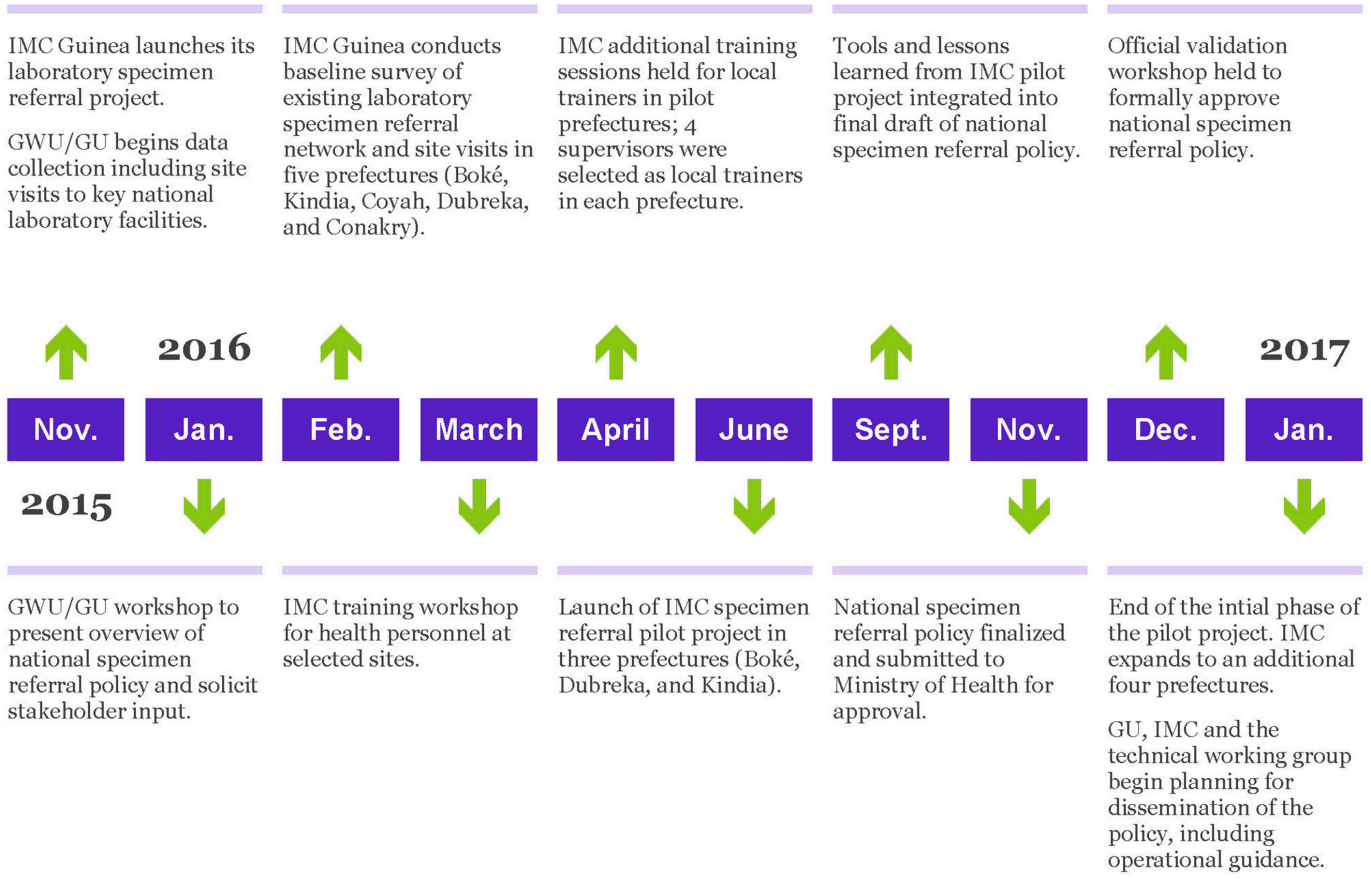
Building the national specimen referral system in Guinea - key project milestones.

A timeline of the project is provided in Figure 1.

### Development of a National Policy for Specimen Referral

The objective of this portion of the project was to support the MOH, with input from other technical Ministries as required, to develop a national reference document that would serve to describe the basic principles of the specimen referral network, based on minimum diagnostic capacities expected at each level of the system and in accordance with existing national guidelines and policies.

#### Stakeholder Engagement and Laboratory Network Mapping

In order to ensure stakeholder input and country ownership of the process, a highly collaborative approach was taken, focusing on the establishment of a small, informal, technical working group to guide the day-to-day drafting complimented by several larger stakeholder workshops to gather input from the broader community of laboratory partners and agencies in Guinea. The technical working group was fluid, based on availability and need, while the core comprised of key individuals from the MOH (Laboratoire National de Santé Publique), the Faculty of Medicine at the University Gamal Abdel Nasser of Conakry, and experts from CDC and GU. This core group was derived from members of the laboratory sub-commission of the National Ebola Coordination Cell (CNLE), which also included other laboratory stakeholders such as IMC, Fondation Mérieux, and Institut Pasteur-Guinée.

The first major step in the project was to review the current landscape of specimen referral and the laboratory system, with reference specifically to Guinea's national health structure, consisting of national, regional (eight administrative regions), prefectural (equivalent to a district; there are 33 prefectures in Guinea, plus five equivalent communes in Conakry), and sub-prefectural (community-level) tiers of capabilities. This effort began in November 2015 through a combination of direct site visits to assess existing systems and structures for specimen handling and referral, as well as consult with key stakeholders. The project team visited four national level laboratories: the Ratoma Ebola Diagnostic Center (REDC), the diagnostic laboratory at Ignace Deen Hospital, the National Public Health Laboratory (LNSP), and the Central Veterinary Laboratory (LCVD), and utilized a standard questionnaire to collect information regarding processes and systems for specimen collection, management, transport, and referral. A copy of the questionnaire is available as Supplementary Material. Additional information was gathered directly from a broad array of stakeholders, including the Ministries of Livestock and Environment, during a large laboratory partners meeting hosted by the MOH and GU in Kindia. Partners were allocated to working groups to address priority issues for laboratory capacity building. One working group focused on the issue of specimen referral, and was specifically tasked with identifying the roles and responsibilities at each level of the laboratory system with respect to specimen collection, storage, transport, and elimination, as well as the anticipated diagnostic capacities required at each level, through adaptation of the Maputo Declaration's recommendations to Guinea's tiered laboratory network. The stakeholders were also asked to provide recommendations on mechanisms or models for integrating specimen collection and transport into Guinea's health system.

#### Designing a Specimen Referral Policy

In addition to mapping national capacities and existing laboratory infrastructure the team reviewed best practices and examples from other countries for core elements that might be adaptable to the Guinean context. The project team focused on examples of successful specimen referral networks from low income countries, and where possible, those that established integrated systems covering multiple diseases, rather than vertically funded programs. The team also reviewed existing national and international frameworks, policies, and regulations that could be relevant to specimen referral; some of these, notably Guinea's National Biomedical Laboratory Policy, were concurrently under revision at the same time as the specimen referral policy was being developed, and thus were not available in final form to provide guidance.

Based on the information gathered during the November 2015 laboratory site visits and partners meeting, as well as follow up discussions with stakeholders via weekly CNLE laboratory sub-commission meetings, the project team developed an outline of a national specimen referral policy. The outline was presented to relevant stakeholders, including technical ministries and partner organizations at a January 2016 workshop. At the workshop, participants were divided into three working groups to refine the policy outline and provide suggestions for content: secure sample transport and collection; sample management (from testing to safe waste disposal); and data management and evaluation.

Between January and November 2016, the project team drafted content for the policy, relying heavily on input and feedback from the informal technical working group and laboratory sub-commission of the CNLE (which met biweekly during this time). Concurrently, input from the outcomes of IMC's pilot implementation project was integrated (please see pilot implementation project described later in this section). A summary document, outlining the objectives and content of the policy, was created to serve as a reference guide and assist in socializing the policy across various stakeholders. The final draft of the national specimen referral policy was formally submitted to the Minister of Health, with copies provided to the Director of the National Public Health Institute (INSP) and the Director General of the National Health Security Agency (ANSS). At the request of the Ministry of Health, in December 2016 GU organized a “Validation Workshop,” to allow for key stakeholders to provide final comments and suggestions. At the conclusion of the validation workshop, the policy was formally approved and adopted by the Ministry of Health (13).

### Pilot Implementation Project

The pilot project was implemented by IMC in three prefectures in Lower Guinea, namely Boké, Dubreka, and Kindia, following a baseline survey of existing referral practices to identify target areas and select sites.

#### Baseline Survey of Existing Referral Practices

In order to select the targeted prefectures for the pilot, IMC conducted a baseline survey of the existing laboratory specimen referral network from January 6 to March 15, 2016. The study focused on evaluating existing specimen collection practices, and the testing and transport capacity of community health centers as well as their prefectural reference laboratories. The study was carried out in the prefectures of Boké, Kindia, Coyah, Dubreka and the capital city Conakry, where IMC had existing projects. These prefectures represent three out of Guinea's eight administrative regions, and thus serves as an approximation of the variation that likely exists between and among different level facilities in the country as a whole. In each of these five areas, IMC selected three community health centers (Center de Santé) to evaluate, prioritizing facilities that MOH officials reported to have functional laboratories and trained laboratory staff. IMC also assessed each area's reference laboratory in order to gain a comprehensive understanding of the specimen transfer system from point of collection to point of diagnostic exam.

#### Implementation of the Pilot Project

**Figure 2:**
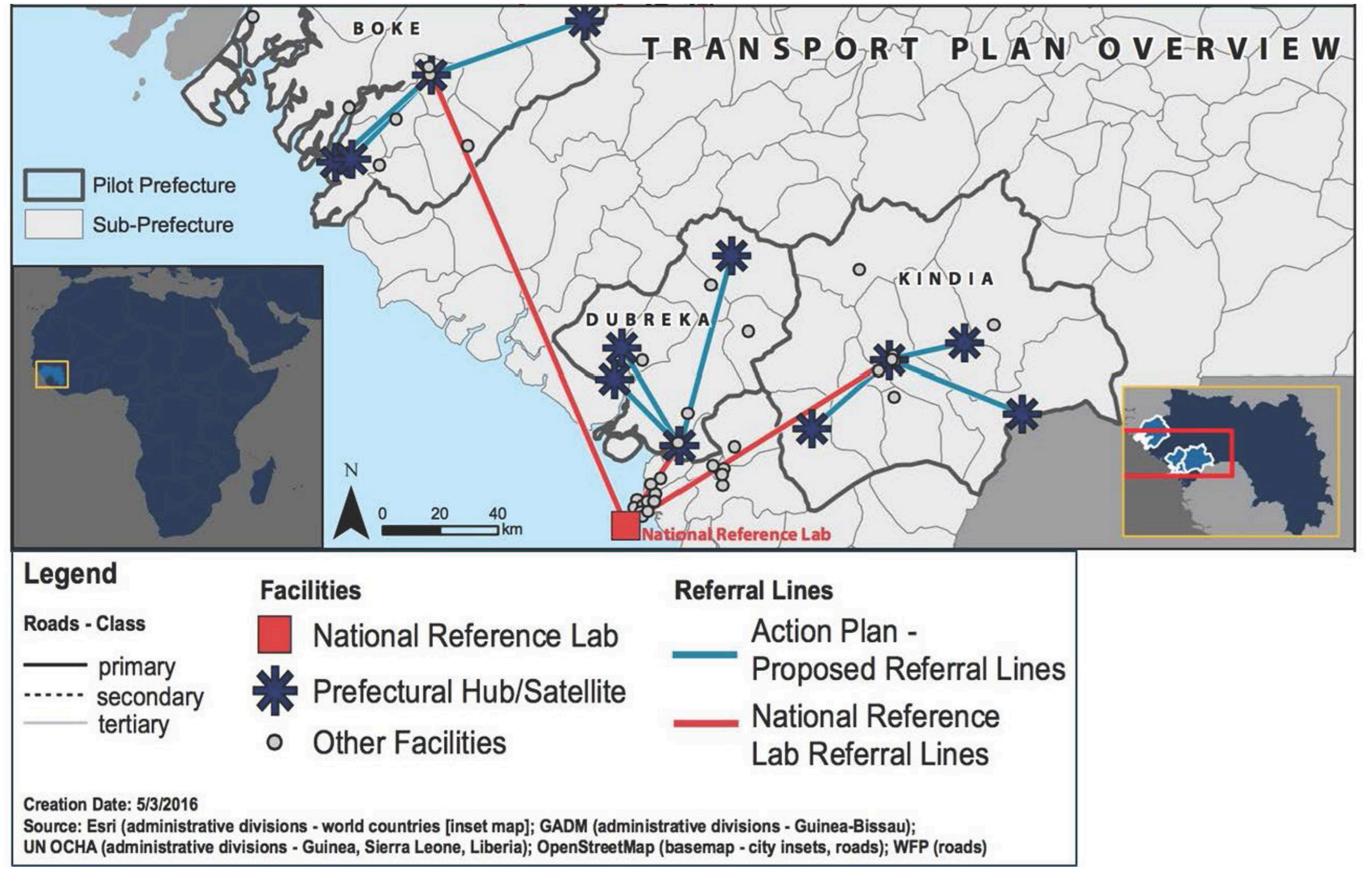
Map showing the prefectures included in the pilot implementation project, including transportation plan overview.

Immediately after the assessment, IMC selected three prefectures to be included in the specimen referral pilot project. Selection criteria included distance from the referral laboratory in Conakry and whether the prefecture included remote health clinics with difficult access. Among the prefectures assessed, Boké, Dubreka, and Kinda were selected. The baseline assessment also provided an opportunity to conduct the mapping of health facilities in the selected prefectures in order to select those which were qualified to serve as central hubs for the pilot specimen referral network, as well as the “spoke” facilities that would refer specimens to each respective. Figure 2 shows a map of the pilot prefectures and proposed referral routes for the pilot project.

Hub facilities received solar refrigerators for specimen storage and both the hubs and peripheral facilities received specimen collection and packaging materials. In addition, IMC implemented a cost reimbursement scheme in each prefecture covering expenses incurred from transporting the samples from the communities to the health centers and from the health centers to the prefectural health department (Département Préfectoral de Santé, DPS).

In order to strengthen laboratory technicians' capacities to safely and securely collect, package, store, and transport biological samples from the community to the health center and prefectural laboratories, IMC worked with the INSP and each DPS to:

1. Organize capacity-building workshops for health personnel and laboratory technicians from the health clinics and the prefectural hospital,
2. Implement simulations based on sampling, packaging, and transport protocols,
3. Organize joint supervision with district health authorities to monitor the activity.

Trained MoH health staff were designated as being in charge of specimen transport during the pilot project in the selected prefectures. In the event a dedicated health workers were not available, an IMC program officer ensured the transportation of samples. Motorbikes were used for specimen transport between the communities, the health centers and the DPS. From the DPS to the regional or national level, vehicles were used for specimen transport. For highly infectious specimens, vehicles were used at all levels.

#### Evaluation of the Pilot Project

IMC developed an assessment tool to assess service availability and service readiness. The tool was used by locally hired Program Officers on a monthly basis to review performance of supported laboratories, assess adherence to specimen transfer protocols, and track progress in specimen transfer capacity strengthening. More specifically, the tool assesses six areas of laboratory performance: (1) adherence to infection prevention and control (IPC) protocols; (2) availability of specimen collection supplies; (3) staff competencies; (4) data management; (5) waste management; and (6) availability of basic amenities, equipment, and supplies (status of the facility, water, and electricity). The tool was used to assess the aforementioned domains in Kindia, Dubreka, and Boké, and cover all health facilities supported through the project. The indicators were designed to be applicable both to the pilot project, as well as for potential long-term implementation.

In addition, IMC conducted a mid-term learning workshop with the MOH and other humanitarian partners where project progress was discussed.

## Results

### National Specimen Referral Policy

The full process to develop the national specimen referral policy took over 1 year, from initial scoping and data collection until final approval and adoption by the Guinean Ministry of Health. The policy represents the culmination of extensive collaboration and coordination among dozens of partners and organizations, and presents a synthesis of international best practices for specimen referral as adapted to the unique circumstances of post-Ebola Guinea and in accordance with national and international frameworks. In order to remain flexible and applicable despite the rapidly changing and improving laboratory capacity within the network, the policy does not contain detailed operational guidance in the main text, but rather describes the general measures to be taken into consideration in collecting and managing samples at each level of the laboratory system, including basic principles for specimen transport, sequential diagnostic testing, protection of sample integrity, biosafety and biosecurity, and monitoring and evaluation. Operational guidance, including standard operating procedures and job aids, will be provided as part of the process of disseminating and implementing the policy across the laboratory network, and may be included as readily editable annexes.

The policy contains the following key elements:

- Establishes roles and responsibilities at each level of the laboratory network in the collection and management of samples, their packaging, shipping, transport, monitoring, storage, or disposal;
- Describes the general measures to be taken into consideration in collecting, transporting, and managing sampling at each level of the laboratory system, including biosafety and biosecurity considerations;
- Provides guidance for determining the necessary resources and timetable for the establishment of the national collection network;
- References the governing bodies required to implement the policy, including responsibilities for organizing, coordinating, funding, and overseeing specimen referral functions; and
- Provides guidance for establishing a system for monitoring and evaluating the flow of samples through the network for continuous improvement.

These elements built on the existing responsibilities of the different tiers of the health system for collecting and transporting specimens, but which had previously not been formally codified, comprehensively defined, nor described in detail. Overall, the policy therefore created one standardized framework with clear expectations and defined responsibilities for each level of the health system, with respect to specimen referral. The summary guide to the policy was disseminated widely after final approval by the MOH, with copies sent to all prefectural health departments and associated hospitals. Working closely with the ANSS and other partners involved in public health workforce development activities, information on the specimen referral policy and its key elements has also been integrated into training modules for the Field Epidemiology Training Program (FETP) as well as surveillance training at the national and prefectural levels.

**Figure 3:**
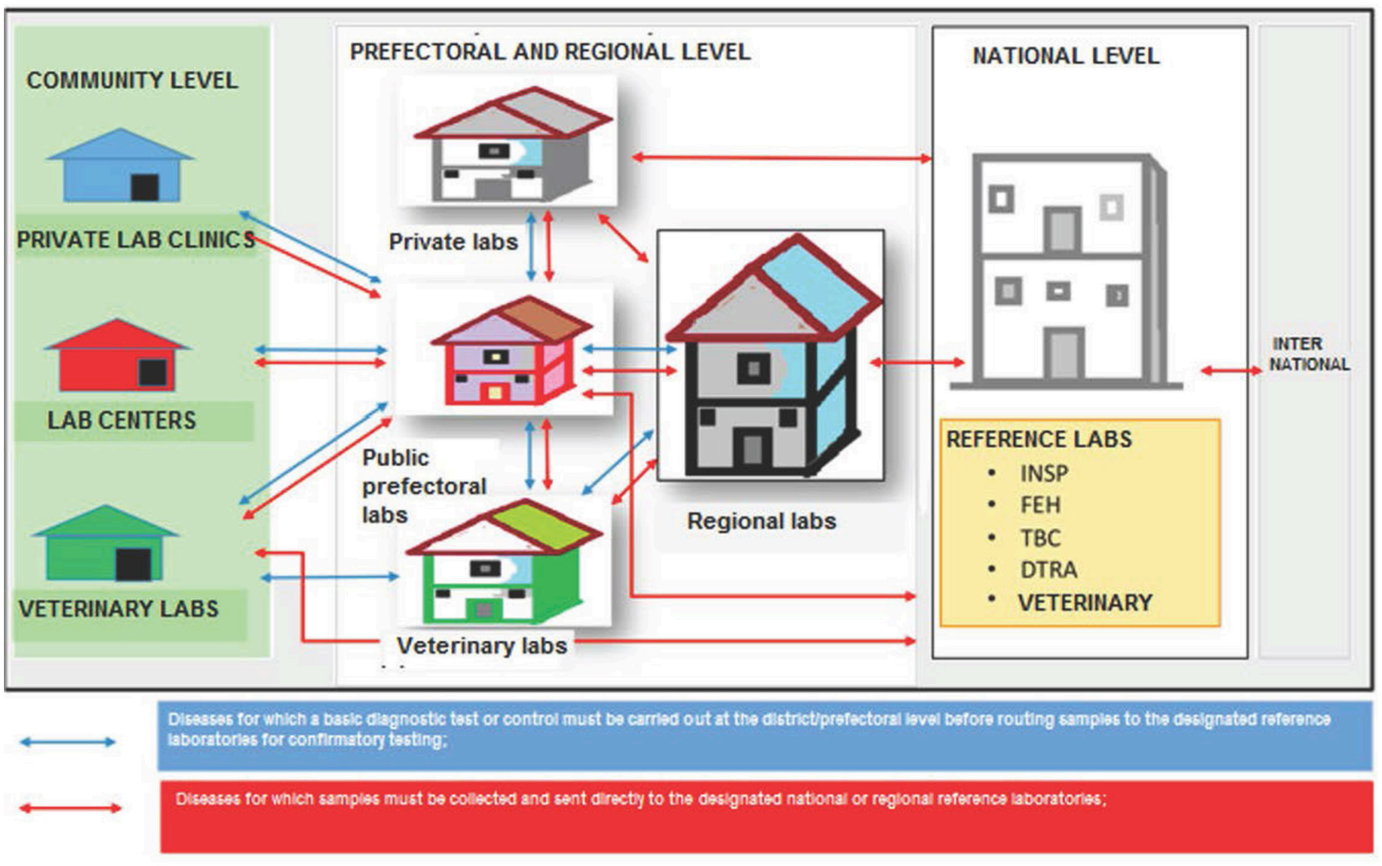
Schematic of “star” transportation model adopted by consensus by stakeholders for referral of specimens from sub-prefectural to prefectural and regional laboratories, and up to the national level. INSP, National Public Health Institute; FEH, National Viral Hemorrhagic Fever laboratory; TBC, National Tuberculosis Reference Laboratory; DTRA, Ratoma Ebola Diagnostic Center.

One key outcome of the stakeholder consultations during the policy's development was the decision to adapt the traditional “hub and spoke” model adopted in Uganda and Haiti (5, 6) to account for factors deemed important in the Guinean context, as well as data points identified during the process of developing the policy (Figure 3). Describing it as a “star,” the consensus was that a formal circuit of transport servicing sub-prefectural health centers and posts might not be cost-effective, and that utilizing vehicles provided at the prefectural health departments to conduct individual visits to sub-prefectures to collect samples would be more efficient. Of note, it was observed that sample numbers will vary widely between prefectures and regions, therefore the policy does not itself establish a schedule for specimen collection and pick-up by each prefecture, but rather requires that pick-ups be established based on local conditions. Similarly, the experience of the Ebola outbreak prompted stakeholders to place a high importance on ensuring the referral network was harmonized with notification requirements, such that specimens from suspect cases of “priority diseases of epidemic potential” (as defined by the MOH and IDSR) would bypass the usual tiers of testing and be sent directly from the prefectural level to the appropriate national reference facility.

In addition, stakeholders agreed it was important to include both private sector laboratories as well as the veterinary sector in the national specimen referral system. This acknowledges the significant role the private sector plays in patient care in Guinea, as well as the country's growing commitment to implementing a “One Health” approach for disease surveillance and response. Moreover, it references the observation from the site visits (Table 1) that diagnostic capacity and particularly international referral capabilities exist at the LCVD, and thus the overall network would be strengthened from inclusion of the veterinary sector, and especially for early detection of zoonotic diseases. In turn, the veterinary sector, which has limited laboratory capacity outside of Conakry, would benefit from a formal system of referral of specimens from its peripheral animal health departments and livestock stations. While the Ministry of Environment is responsible for monitoring of wildlife populations, particularly in Guinea's protected areas, and is therefore an important stakeholder, currently it has extremely limited laboratory infrastructure and no testing capacity for infectious diseases.

**Table 1:** Summary data from site visits to national laboratories.

**Lab name**	**Are samples received from other levels of the health system?**	**Are samples collected on site?**	**If the required diagnostic test is not available, is the specimen referred?**	**Is there a central point for specimen receipt?**	**How are samples inventoried and stored?**	**How are samples packaged for transport?**	**Are SOPs available for sample receipt, management and/or transport?**
Ratoma Ebola Diagnostic Center (REDC)	Yes, samples are received from different levels of the health system and various geographical locations	No	No. Lab only tests for Ebola virus, and all required equipment and reagents are available on site	Yes. There is one box for sample receipt. During EVD outbreak, samples dropped off by Red Cross volunteers	Samples are recorded on a standard INSP form and are destroyed after testing (incinerator on site)	Not applicable; samples are not transported	Yes
National Public Health Institute (INSP)	Yes, sometimes from prefecture labs	Yes, the majority of samples are collected on site	Yes—usually referred to Dakar (Senegal)	Cholera and HIV samples are taken straight to the respective diagnostic laboratories. There is a central reception on the main floor	HIV samples are databased by patient number and stored on site. Cholera samples indexed as part of AFRICHOL project and archived in South Africa	Triple packaging; laboratorians have been trained in IATA specifications	No
Ignace Deen Hospital	Yes, occasionally meningitis samples received from regional lab	Yes, the majority of samples are collected on site	Yes—if tuberculosis (TB) is suspected, sample is sent to the national TB reference lab. All other specimens are sent to INSP	There is a central repository for samples collected on site but no standard process for receiving samples from elsewhere	Patients complete a form which is attached to the sample during testing. Samples are usually destroyed after testing (there is an incinerator on site)	Single packaging. Packaging materials are restocked from France	No
Laboratoire Central de Diagnostic Vétérinaire (LCDV)	Yes, samples are received from the field (such as prefectural livestock departments)	No, but teams from the LCDV will go into the field to take samples and bring them directly back	Yes—have relationships with a number of international reference labs, including in France, the UK, the US, and Senegal	Yes, there is a central receiving office for samples	Sample information is recorded on paper; aliquots are given ID numbers. Most samples stored in freezers for long time. Negative samples may eventually be destroyed	Triple packaging for international transport. FAO provided IATA training to staff	No

### Pilot Implementation Project

#### Baseline Survey Findings

IMC's baseline assessment survey revealed that no formal system existed in any of the areas of study that would allow community health centers to refer and transport samples to prefectural or regional laboratories. For suspect cases of epidemic diseases, the prefectural or communal health director (Directeur Préfectoral/Communal de la Santé) or the doctor in charge of disease (Médecin Chef des Maladies, or MCM) was traveling directly to the patient, collecting the sample, and was personally dispatching it to the National Reference Laboratory in Conakry. The costs of transport were then reimbursed by WHO. For samples that required long-distance transport, for instance from Boké to Conakry, WHO was only reimbursing transport fees but not the accommodation or other expenses incurred while traveling overnight. During the Ebola outbreak, a parallel system emerged: for all community deaths and suspect cases of Ebola, the Guinean Red Cross was responsible for collecting and transporting the sample to the nearest PCR-equipped laboratory.

None of the community health centers assessed were regularly sending laboratory samples for referral testing. In the prefectures of Boké, Kindia, Coyah, and Dubreka, if a patient needed routine laboratory testing that was unavailable at the health center, the patient was referred directly to the prefectoral or regional laboratory, in certain cases hours away and difficult to access due to limited, *ad hoc* transportation. In Conakry, samples were also infrequently transported between health centers and the referral laboratory, and then only for specimens of suspected diseases of epidemic potential, and limited to when health center staff were available to personally ensure the means of transport (via vehicle or walking). These findings were consistent with later national-scale assessments of laboratory capacity, undertaken by Fondation Mérieux via the LAB-NET project; IMC's data were integrated into the final map of the national laboratory network (14).

In all areas, lack of basic infrastructure and equipment hampered efforts to provide sample collection, storage, and testing and referral services at the community health center level. While rapid diagnostic tests for malaria and HIV were consistently available at all health centers visited, provided by the respective vertical disease control program and funded by external partners, other laboratory services were dependent on the health center staff's personal ability to personally procure reagents and tests.

Clinicians at community health centers informally reported receiving little to no training on sample collection, storage, referral and reporting in the last year and prior to the EVD outbreak. The few trainings provided had been part of general IPC curricula implemented as part of the Ebola response. Therefore, the information focused on laboratory activities that was provided during these trainings was fragmented and superficial, and largely failed to address relevant sample management issues from a holistic perspective.

Finally, standardized systems for supervision and quality control were found to be non-existent. Nearly all laboratories noted difficulties in restocking specimen collection and transport materials, and less than a third had a pre-defined system that enabled them to keep track of the average specimen transfer or referral time.

#### Project Outcomes and Training

Working in close collaboration with the ANSS and MOH, IMC successfully launched the pilot program in three prefectures, and demonstrated success in dramatically shortening the time needed to collect, process, and transport blood samples and other diagnostic specimens (see Tables 2, 3 below).

**Table 2:** Number of specimens transported during the pilot implementation project, by disease and type.

**Disease**	**Type of specimen**	**Prefecture**			**Totals**
		**Boké**	**Kindia**	**Dubreka**	
Ebola virus disease (EVD)	Buccal swab	0	0	98	98
	Vaginal secretion	15	0	0	15
	Sperm	10	0	0	10
	Breastmilk	2	2	0	4
Acute flaccid paralysis (AFP)	Stools	39	13	21	73
Measles	Blood	5	34	60	99
Yellow fever	Blood	25	9	3	37
Cholera	Stools	0	0	1	1
Meningitis	Cerebrospinal fluid	0	0	2	2
Total		96	58	185	339

**Table 3:** Assessment data from the first year of the pilot implementation project.

**Indicators**	**Numerator**	**Denominator**	**Percentage achieved (%)**
% of health facilities visited that complies with IPC protocols	12	15	80
% of health facilities at which lab technicians demonstrate strong theoretical knowledge of specimen collection, packaging, storage, and safe transport	15	15	100
% of health facilities having adequate data management system	8	15	53
% health centers fully adhere to waste management protocols	12	15	80

In collaboration with the INSP and Fondation Mérieux, IMC staff developed training curricula for safe specimen collection, packaging, and transport in reference to the Guinea National Policy for Specimen Referral developed by GU and the MOH. These training materials complement existing IDSR curricula produced by WHO, and have been indispensable in ensuring high quality trainings. In total, 236 health center staff were trained during the pilot implementation project, including 68 from Dubreka, 92 from Kindia, and 76 from Boké, held across two training sessions. IMC also had the opportunity to send a Program Officer from the pilot project to a biobank training conducted by Fondation Mérieux's LabNet program in Morocco in February 2016. Upon their return, the Program Officer replicated the training for staff working on the pilot implementation project in Guinea, and utilized the materials to enhance further training sessions for health center staff.

#### Specimen Referral Metrics

Between February 2016 and January 2017, 339 specimens were referred from the collection point to the nearest suitable laboratory, as defined by the national policy. These specimens included 127 suspect EVD samples, 99 measles samples, 73 acute flaccid paralysis (AFP) samples, 37 yellow fever samples, 2 meningitis samples, and 1 cholera sample.

Table 2 summarizes the number of specimens transported by type.

The average transport cost was 67,500 GNF (~7.5 US dollars/specimen, as of January 2017) with a standard deviation range of 2,800 GNF. The average time for the specimen transport between the communities and the health clinic to the receiving laboratory of 14 h with a standard deviation of 11.7 h. All the specimens received were of good quality at the time of their reception at the referral laboratory.

#### Evaluation Results

The main findings from assessments conducted throughout the first year of the pilot project are summarized in Table 3.

Overall, there was good compliance with occupational safety and biosafety protocols across the health facilities visited, with 80% demonstrating full compliance with IPC measures and the same proportion fully adhering to waste management protocols. All laboratory technicians evaluated were able to demonstrate strong theoretical knowledge of specimen collection, packaging, storage, and safe transport. However, some shortcomings were observed: data management remains an area requiring improvement, with almost half of the facilities lacking adequate systems for accurate and timely data collection, entry, and storage. Across all sites, it was also observed that the lack of or inconsistent availability of basic amenities, including water and electricity, for the full duration of opening hours prevented certain health facilities from performing laboratory tests on site or storing samples as needed

IMC Program Officers addressed specific issues with staff from each laboratory assessed and made recommendations for improvement. Recommendations made during supervisions included: store used security boxes in a secure location before their transportation to the prefectural hospital for incineration; use 0.05% chorine solution for disinfection; display handwashing posters in the laboratory; and correctly use the inventory cards in the laboratory.

According to participants from the DPS and Guinea's laboratory network who attended IMC's mid-term learning workshop, the main achievements of the project were increased specimen collection, packaging and storage capabilities and the increase in specimen referral and transport processes as a result of the project.

## Discussion

This paper serves to describe the process of developing a national specimen referral system in Guinea, focusing on the challenges and opportunities associated with building sustainable capacity in the wake of a major public health crisis. It important to note that the observations described in this paper should be taken as preliminary lessons learned in what will be a long-term process.

One of the initial and most significant challenges faced by the project was that it started while the Ebola response was still underway. While, by the time data collection began in earnest, case numbers had dropped to zero, the priorities of the Guinean government remained firmly on Ebola “Phase III” surveillance, which supported continued enhanced case finding and community reporting to break any remaining chains of transmission and ensure no new flare-ups of disease (15). Despite these efforts, mid-way during the project, in March 2016, a cluster of cases was reported from the Forest Region, diverting attention once again from recovery and long-term capacity building and back to emergency response procedures and actions. As such, one general lesson that may be applicable to other settings is that any interventions that seek to strengthen specimen referral systems during or immediately after major outbreaks should ensure that guidelines for sample collection and transport are aligned with response messaging, and designed such that they can integrate all pathogens without disrupting ongoing surveillance efforts. Leadership from the MOH and other authorities is critical in ensuring that support is provided in the transition from response to recovery, and that response activities are not considered “separate” from longer-term capacity building.

Another obstacle with respect to developing an effective and sustainable specimen referral system is the requirement for coordination in establishing a fully integrated and tiered laboratory system, with facilities and staff equipped and trained at each level of the network to manage samples appropriately, safely, and securely. Guinea's laboratory system was severely under-resourced prior to the outbreak. During the EVD crisis, donor assistance was not always provided in line with the national health pyramidal structure, meaning that post-Ebola, there is a wide discrepancy between the infrastructure, personnel, and capabilities across laboratories even at equivalent levels (prefecture, regional, national) within the network. For example, some prefectural laboratories were provided with advanced PCR-based diagnostic equipment, while these machines are not yet available at all regional laboratories. Similarly, several mobile laboratories were deployed to geographic regions where diagnostic needs were the most urgent, while other more permanent laboratory structures were provided by donors to locations justified less by need than by space or political considerations. The four mobile laboratories in Lower Guinea that remained after the outbreak—the REDC, the two K-Plan laboratories, and the CREMS facility—are located in Nongo (a neighborhood of Conakry), Kaloum (on the grounds of Ignace Deen hospital, also in Conakry), near the prefectural health department in Forécariah, and at the former site of the “Pastoria” facility in Kindia, respectively. The latter now boasts a biosafety level 3 facility, the first of its kind in Guinea (12); all four facilities, plus another laboratory in Nzérékoré supported by the Viral Hemorrhagic Fever Project of Guinea (PFHG), possess advanced PCR diagnostic capabilities yet are not formally considered reference laboratories, and it remains unclear how these high-capability laboratories will be incorporated into the national laboratory network, including their potential role in specimen referral and confirmatory testing.

Overall, the large number of partners, projects, and activities operating in Guinea during and after the outbreak led to a number of challenges, not least that key decision-makers within the government were often stretched across multiple responsibilities and priority engagements. The necessity of ensuring that all stakeholders were able to provide input and contribute to the development of the national policy delayed its finalization, but resulted in a more robust and implementable final product that was readily adopted by those it will impact.

On a positive note, the National Specimen Referral Policy has received strong endorsement from authorities and stakeholders guiding other policy efforts. Since 2014, a process had been underway to revise Guinea's National Biomedical Laboratory Policy, which has the mandate to define and codify the structure and function of the national laboratory network. An updated version was validated by the MoH in early 2018; the new National Biomedical Laboratory Policy references the National Specimen Referral Policy developed during the course of this project, cementing its position as a key document within Guinea's laboratory system and enhancing the long-term sustainability of its application.

Based on the successes of the implementation of the pilot project, IMC received additional funding from CDC in February 2017 to expand the specimen referral network to four new prefectures, namely Coyah, Kissidougou, Gueckedou, and Macenta. As of November 2017, implementation of the specimen referral network is underway in these four new prefectures, while IMC continues to maintain the system in the three pilot prefectures, for a total of seven prefectures. The technical working group, established at the outset of the project, continues to provide guidance on the implementation of the project, including expansion of the network to new prefectures, as well as dissemination and raising awareness for the policy itself. These steps are part of the process in transitioning the management and implementation of the specimen referral system fully to the Ministry of Health, a critical step for future sustainability.

Indeed, one of the next steps, after adoption of the policy, was to develop and disseminate operational guidance in support of the policy, for use by all partners and stakeholders, across all levels of the laboratory and health systems, to “universalize” specimen referral nation-wide. A set of diagnostic job aids as well as specimen collection and packaging standard operating procedures (SOPs) were developed by GU and trialed at a training workshop co-hosted by GU, IMC, and the MOH in January 2018. Feedback from the participants, representing laboratory and surveillance focal points for each of the communal medical centers in Conakry, was very positive, and as of February 2018, plans are underway to disseminate the job aids and SOPs more widely.

Provision of further training and resources for safe and secure implementation of the policy will also be critical, and a long-term sustainability challenge. To achieve this, activities related to specimen referral must be implemented into future strategic planning efforts by the MOH. This must include consideration of funding; eventually, the system should be fully supported financially via budget lines from the MOH, though support from external partners is justified initially, and particularly during expansion throughout the country. Aligning national specimen referral with existing supply chain networks for vertical disease programs, such as malaria or HIV, should be explored further with the relevant partners. As observed during the pilot implementation project, provision of basic amenities, such as water and electricity, cannot be taken for granted. Initial expansion will focus on ensuring that the policy is implemented throughout the public sector's laboratories, down to the health center level, but eventually, such activities will have to extend to the private sector and animal health laboratories. Indeed, to further encourage Guinea's efforts to institutionalize One Health within their surveillance and laboratory systems, integration of environmental laboratories, which to date remain extremely weak, into the national network should be a priority. Too often, funding for capacity is siloed by discipline, and excludes the private sector. Fortunately, some cross-cutting programs are being established in Guinea which are able to at least provide support jointly to the human and animal sectors; examples include USAID's Emerging Pandemic Threats program (16, 17), and the World Bank's REDISSE initiative (18). Unfortunately, beyond integration of wildlife surveillance, neither program provides direct support for developing laboratory capacity for environmental health, nor integrating environmental health samples into the national referral network.

Finally, implementation of the national specimen referral system on a country-wide level must include a robust and comprehensive monitoring and evaluation framework. The framework must include assessment of the function of the referral network, including time for transport and receipt of samples and reporting of results, as well as tracking of costs. Building on the indicators established by this pilot project would help ensure continuity of monitoring, and allow for progress to be measured against an existing initial baseline. In addition, indicators should be aligned with the broader laboratory quality assurance program concurrently being developed in Guinea, by the Association of Public Health Laboratories (APHL) in collaboration with the MOH, with funding from CDC. As familiarity with the specimen referral policy grows, and more samples are referred through the system, the MOH could also consider rolling out mobile-enabled data management systems, perhaps linked to the DHIS 2 electronic system already in place for surveillance at the prefectural level. As of March 2019, only the national reference laboratory has been integrated with the DHIS 2 platform for reporting diagnostic results; in the future, this could potentially be extended throughout the laboratory network.

Eventually, a cost-effectiveness analysis should be undertaken, similar to that performed by Uganda when it reformed its laboratory sample transport system to improve HIV testing (3). This will facilitate future budgeting by the MOH, and allow for transition of costs from external partners to the national government, while also potentially identifying areas for further cost savings as well as encourage economies of scale in the system. Presently, given the low overall volume of specimens, the cost per specimen is acceptable only for high priority samples. Coordinating these various efforts, each of which is interlinked and co-dependent on other activities, will be a challenge. Sustainability of the resultant system must also remain at the forefront of any efforts to build laboratory capacity in Guinea. Guinea's experience in developing a fully integrated national system for specimen referral can provide useful guidance and lessons learned to other countries planning similar efforts, both to date and as it continues to implement the system in the future.

## Author Contributions

CS, RM, JF, WH, LK, SL, ES, AV, AD, and LM conceived the project and developed the methods. CS, MB, AB, EB, JF, LK, SL, ES, AV, and AD contributed to the development and finalization of the specimen referral policy and drafted those sections of the manuscript. RM, MB, EB, WH, LK, SL, AV, and AD contributed to the design, execution, and evaluation of the pilot implementation project and drafted those sections of the manuscript. CS, EB, ES, and LM drafted the introduction and discussion, and all authors reviewed and approved the final manuscript.

### Conflict of Interest Statement

The authors declare that the research was conducted in the absence of any commercial or financial relationships that could be construed as a potential conflict of interest.

## Footnotes

^1^Until June 2016, the GU team were based at the George Washington University (GWU).
