# Supplementary material for: Creating a National Specimen Referral System in Guinea: Lessons From Initial Development and Implementation
Source: Front Public Health. 2019 Apr 16;7:83. doi: 10.3389/fpubh.2019.00083 (PMC6499205; doi:10.3389/fpubh.2019.00083)
Supplement: Supplementary file 1 [file Data_Sheet_1.PDF]

Visites de laboratoire - Acheminement des échantillons « Une Seule Santé »

Laboratory visits - "One Health" specimen referral

Recrutement *Staffing*

1) Combien de personnel avez-vous? À temps plein ou à temps partiel?
   How many staff do you have? Full-time or part-time?

SOP

1) Y at-il SOP (PON) sur place pour: *Are there SOPs in place for:*
   a. La collecte et le transport d'échantillon (ce que pour recueillir, la façon de recueillir et où l'envoyer à)?
      The collection and transport of samples (who/how to receive, to whom to send)?
   b. Les testes diagnostics de laboratoire (touts les tests effectués)?
      The diagnostic tests (all those performed)?
   c. Rapports de laboratoire?
      Laboratory reports?
2) Y a t-il une formation régulière pour tout le personnel? La formation est facilement accessible à tout le personnel? À quelle fréquence?
   Are all staff regularly trained? Is training easily available to all personnel? How often?

Le traitement des échantillons *Sample management*

1) Comment sont les échantillons reçus? *How are samples received?*
   a. Avez-vous reçu généralement échantillons visés à partir des laboratoires de la préfecture? Recevez-vous des spécimens ou des échantillons provenant d'autres sources?
      Do you generally receive samples from the prefectural laboratories? Do you receive samples from other sources?
   b. Vous recevez uniquement des échantillons cliniques / patient pour les tests de diagnostic initiales, ou vous recevez également prélèvement / échantillons visés pour des tests de confirmation?
      Do you only receive clinical/patient samples for initial testing, or also samples requiring confirmatory tests?
   c. Est-ce que le laboratoire a établi les exigences d'emballage prélèvement pour recevoir des échantillons? Si non, comment les échantillons sont reçus, généralement?
      Has the laboratory established packaging requirements for receipt of specimens? If not, how are samples generally received?
   d. Y a t-il lieu central où les échantillons sont reçus et répertoriés avant de tester?
      Is there a central location where samples are received and inventoried before testing?
   e. Quelle est la prochaine étape après réception?
      What is the next step after being received?

f. Comment sont inventoriés échantillons? Y a t-il une personne en particulier (s) responsable de l'inventaire de l'échantillon?
How are the samples inventoried? Is there one person who is specifically responsible for sample inventory?

2) Quels sont les tests de diagnostic que vous exécutez dans le laboratoire?
Which diagnostic tests do you perform in the laboratory?
   a. Quels sont les tests les plus communs?
   Which are the most common tests?

   b. Avez-vous des réactifs et du matériel suffisant pour exécuter les tests nécessaires, ou d'un financement pour maintenir les fournitures nécessaires à des niveaux un problème?
   Do you have sufficient reagents/materials to perform the necessary tests, or funding to maintain sufficient supplies if there is a problem?

3) Quels sont les pathogènes les plus communs détectés?
Which pathogens do you detect most commonly?

4) Y a t-il les modes opératoires normalisés [SOP] pour les tests de diagnostic disponibles et à jour pour chaque niveau de laboratoire?
Are SOPs for all routine diagnostic tests available for each step and laboratory?

5) Y a t-il des tests de diagnostic standard d'or définis pour chaque maladie prioritaire dans le laboratoire?
Are gold standard diagnostic tests defined for each priority disease in the laboratory?

6) Pouvez-vous tester pour: *Can you test for:*
   - ◻ Rage *Rabies*
   - ◻ Charbon bactériale *Anthrax*
   - ◻ Brucellose bovine *Bovine brucellosis*
   - ◻ Fièvres hémorragiques Virales (FVR, Ebola, Marburg, Lassa, etc) *Viral hemorrhagic fevers*
   - ◻ Trypanosomiase *Trypanosomiasis*

7) Si vous ne possédez pas le diagnostic que vous pensez que vous en avez besoin, ou vous avez besoin d'une confirmation supplémentaire, où envoyez-vous l'échantillon suivant?
If you do not have the diagnostic required, or need additional confirmation, where do you send the sample next?

8) Y a t-il une chaîne de traçabilité pour les échantillons comme ils vont de la préfecture aux laboratoires nationaux? Et pour les laboratoires de référence internationaux?
Is there a chain of custody for the samples as they are transported from the prefectural to the national laboratories? And for international laboratories?
   a. Où sont les échantillons envoyés?
   Where are the samples sent?

b. Est-ce que une liste de laboratoires de référence (interne / international) a été publiée et diffusée à tous les laboratoires?
   Has a list of reference laboratories (national/international) been published and disseminated to all laboratories?
c. Est-ce que chaque laboratoire possède une copie ou une carte de laboratoires de référence avec des informations de contact et les adresses?
   Does each laboratory have a list or chart of contact information for relevant reference laboratories?
d. Comment sont envoyés les échantillons?
   How are the samples sent?

e. Est-ce que quelqu'un suit si les spécimens sont reçus et traités au laboratoire de référence?
   Does someone follow up in the specimens are received and tested at the reference laboratory?
f. Comment sont les résultats de tests de diagnostic communiqués au retour au ministère / officielle qui les a soumis? Et dans quel délai généralement?
   How are the test results communicated to the ministry/official who submitted them? And how long does it generally take?

9) Quel équipement est disponible pour une utilisation? *Which equipment is available for use:*

◻ Machine PCR	Nº. :	*PCR machine*
◻ Incubateur (CO2)	Nº. :	*Incubator (CO2)*
◻ Incubateur (non-CO2)	Nº. :	*Incubator (non-CO2)*
◻ Autoclave	Nº. :	*Autoclave*
◻ Centrifugeuse	Nº. :	*Centrifuge*
◻ Frigo (4C)	Nº. :	*Fridge (4C)*
◻ congélateur (-20 ° C)	Nº. :	*Freezer (-20C)*
◻ congélateur (-80 ° C)	Nº. :	*Freezer (-80C)*
◻ lecteur ELISA et la rondelle	Nº. :	*ELISA reader and washer*
◻ microscope optique de base	Nº. :	*Basic optical microscope*
◻ biosécurité Cabinet	Nº. :	*Biosafety cabinet*
◻ micropipets calibrés	Nº. :	*Calibrated micropipettes*

10) Est-ce que le système de laboratoire ont une LQMS ou AQ / QC normes similaires de l'ensemble du système?
   Does the laboratory system have a LMQS or QA/QC similar to the overall system?

Communication

1) Quel est le temps de demi-tour prévu pour l'achèvement et la communication des résultats de diagnostic?
   What is the expected turnaround time for completion and communication of diagnostic results?

2) Avez-vous / comment communiquez-vous les résultats des tests de diagnostic pour la soumission / demande individuelle / unité? (fax, sms, papier, téléphone)

   How do you communicate the diagnostic test results? By submission/individual request/unit? (fax, SMS, paper, telephone)

3) Est-ce vous communiquez les résultats des tests (positifs ou négatifs) au niveau national? *Do you communicate the diagnostic test results (positive or negative) to the national level?*

   a. Si oui, quand et comment?

      If yes, when and how?

4) Y a t-il une politique (formel ou informel) pour la communication à d'autres laboratoires du secteur? E.g., si la maladie que vous identifiez est zoonotique, est-il un processus pour assurer que MoH, MoEl, et MoEn ou leurs filiales sont chacune en contact?

   Is there a policy (formal or informal) for communication to laboratories in other sectors? E.g. if the identified pathogen is zoonotic, is there a process for contacting the MOH, MOEl, or MOEn?

Le traitement des déchets

1) Comment les déchets de laboratoire traitée? *How is laboratory waste treated?*

   a. Y a t-il un autoclave de fonctionnement disponibles sur le site? (Si oui, est-il régulièrement utilisé avec des indicateurs de performance, tels que les bandes autoclaves?)

      Is there a functional autoclave available on site? (If yes, it is regularly used, with performance indicators, like autoclave tape?)

   b. Y a t-il un incinérateur de fonctionnement sur le site?

      Is there a functional incineraor on site?

2) Sont des échantillons traités et éliminés après le test?

   Are the samples treated and eliminated after testing?

   a. Si oui, comment?

      If yes, how?

   b. Si non, ce qui arrive à des échantillons?

      If no, what happens to the samples?
